# The Work of the Medical Branch

**Published:** 1919-10-25

**Authors:** 


### THE MINISTRY OF PENSIONS.

### I. The Work of The Medical Branch.

The work of the Medical Department as it concerns pensioners consists of:

1. Examining them to see if they are suffering from a disability.
2. Deciding if any disability found is due directly, or indirectly, to the man's service in the Forces.
3. Estimating the amount by which the disabled man is handicapped—from a social as well as an industrial point of view.
4. Deciding if the man's disability can be lessened, or removed, by any form of treatment.

Ultimately the Medical Department will also be responsible for providing accommodation in hospitals and institutions for all pensioners requiring treatment.

The machinery for carrying out these various duties is as follows:

Pensioners are examined by a "Board" of three doctors—a chairman and two members. The man is brought before the Board, undressed if necessary, and is then questioned and gone over in detail by all the ordinary methods of a medical examination. In special cases the man is referred to specialists, who may either be summoned to sit on the Board, or who send a report of their examination to the Board to help them in arriving at a conclusion. For instance, a man who is suspected to be suffering from malaria is sent to have his blood tested by a bacteriologist. A man supposed to have Bright's disease is sent to have his water thoroughly examined. A man with eye or ear disease is seen by an ophthalmic or aural surgeon. A case of heart disease that presents any unusual features or difficulties is referred to a special Board composed of heart specialists: the same thing applies to cases of nervous disease and tuberculosis. While examining a man the Board has before it all documents relevant to the case—*i.e.* the man's "medical history" during his service, his "attestation form" to show what his condition was on joining up, his record of service which tells the Board the kind of work he has been doing—front line, transport, Labour Corps, work at the base, etc.

The policy of the Ministry, as laid down in their instructions to members of Medical Boards, is to treat the pensioner sympathetically and to regard him as one who has suffered in serving his country and not as a malingerer or one whose statements are not to be relied upon: in all cases *the pensioner is given the benefit of the doubt.*

### 1. Constitution of Medical Boards.

The Boards are composed of medical men drawn from the district in which the pensioner lives, so that in many cases they have the advantage of having known him before the war. The members of the Boards are taken from a panel made by the Medical Officer of the Ministry of Pensions who is in administrative charge of the district (Deputy Commissioner of Medical Services). This panel is formed on the advice of the leading doctors of the neighbourhood with the help of such bodies as the British Medical Association. All demobilised R.A.M.C. officers, and especially those with oversea service, are asked to serve on these Boards, and are kept on them unless found, after trial, to be inefficient. *Demobilised officers are always given preference for employment by the Ministry* over those who have not served. All Chairmen of Boards have to be approved by the Director-General, or his representative, before their appointment is confirmed. The Director-General is guided in making these appointments by the reports of the Deputy Commissioners and by a form which has to be filled in by all applicants for employment showing details as to service during the war, qualifications and experience, age, etc. In some districts there is not a sufficient number of demobilised officers to form a panel, and in such a case it is necessary to employ any suitable doctor—irrespective of service. In some places it may happen that the best men in a town are those who are too busy with their own practices to afford the time necessary to serve on Pensioners' Boards: this cannot be avoided.

### 2. The Cause of Disabilities.

One of the duties of the Medical Boards is to decide whether a man's disability is due to his service or has been aggravated by it. As examples of the former wounds may be quoted, or tuberculosis developed for the first time on active service. As an instance of the latter one might take the case of a man with varicose veins on enlistment whose military duties have obliged him to stand for long hours at a time. If the varicose veins are troubling the man when he is discharged, or later, his service would be held to have "aggravated" his disability, on the assumption that he is in worse condition than on enlistment. The Medical Board expresses an opinion in the first place on these points but their conclusions may be altered by the *Medical Assessors*, or, if the man appeals against the decision of the Board or Assessors, or both, the man appears before an Appeal Tribunal. These consist of a President—a barrister—and two members—one sailor and one soldier. There is also a doctor attached to each Tribunal to advise on technical questions. The decision as to whether a disability is "attributable" or "aggravated by" service is really more of a legal than medical question in many cases.

### 3. Assessment of Disability.

The estimation of the *amount* of "disability" incurred by a pensioner is a most difficult problem. Not only has a man's wage-earning capacity to be taken into account, but also his loss from a social aspect; for instance, a man's favourite recreation may be playing the violin; if he has lost a finger of his left hand his ability to earn his own living will not be seriously impaired in all probability but, from the point of view of social amenities, he will have suffered a severe injury.

The "assessment" is made, in the first instance, by the Medical Board which sees the pensioner, but their

**The Ministry of Pensions**—(*continued*).

assessment was in the past liable to be raised, or lowered, by the "Assessors" at Chelsea.

It should be explained that pensions are of two kinds. The ordinary pension is assessed on the basis of a man's "physical capacity" as compared with other normal men: in this case the pensioner's previous occupation and earnings are not known generally, and anyhow are never taken into account. The other kind of pension is known as an "alternative pension" and is based on the pensioner's previous earnings.

Until recently the "Assessors," who are also doctors, have all been stationed in London; they have *not* seen the pensioners themselves but have arrived at their conclusions from documentary evidence only. This procedure has been considerably criticised—and with reason—for it is obviously very difficult in many cases to estimate a man's condition accurately without seeing him—*e.g.*, cases of facial disfigurement, though a photograph is obtained whenever possible in such circumstances.

Also, only a comparatively small number of cases were referred to the Assessors for their opinion: the proportion being dependent on the views of the lay "Award" officer to whom the findings of the Medical Board were sent from every part of Great Britain. Under the new decentralisation scheme, which has already been carried out in some districts, *all* the documents will be seen by the Assessors, who will be situated at the different headquarters—instead of all being in London—and they will not alter the assessments of the Medical Boards, but will refer any cases that they think should be altered to the Board concerned for reconsideration. If the Board cannot see its way to alter its original decision, the pensioner will be brought before a special "Appeal" Board consisting of a Deputy Commissioner, a specialist in the particular type of case—heart, neurological, orthopædic, etc.—and an Assessor. This should, when universally adopted, be a great improvement on the old system, and will do away with the aforesaid criticism as to the alteration of assessments by men who have not seen the case personally.

4. Treatment.

The Medical Board which sees a pensioner can, if it is considered necessary, or desirable, recommend that he shall receive treatment at a hospital, sanatorium, or other institution; but the responsibility for arranging the treatment rests with the Local War Pensions Committee. The Boards can also order certain surgical appliances for a pensioner, such as boots, trusses, elastic stockings, etc.

The Ministry of Pensions is in the process of taking over many of the "war" hospitals established all over the country for treatment of wounded sailors and soldiers; when this has been completed the accommodation will be available for pensioners needing treatment. These hospitals will be staffed by doctors employed and paid by the Ministry of Pensions and will, in many instances, have the same staff as when they were war hospitals.

Blue Tabs.

(*To be continued.*)

